# NCAPD2 is a novel marker for the poor prognosis of lung adenocarcinoma and is associated with immune infiltration and tumor mutational burden

**DOI:** 10.1097/MD.0000000000032686

**Published:** 2023-01-20

**Authors:** Zihao Li, Yuxuan Zheng, Zuotao Wu, Ting Zhuo, Yongjie Zhu, Lei Dai, Yongyong Wang, Mingwu Chen

**Affiliations:** a Department of Cardio-Thoracic Surgery, The First Affiliated Hospital of Guangxi Medical University, Nanning, Guangxi, China; b Department of Respiratory Medicine, The First Affiliated Hospital of Guangxi Medical University, Nanning, Guangxi, China.

**Keywords:** cell cycle, immune checkpoint, immune infiltration, lung adenocarcinoma, NCAPD2, prognostic value, tumor mutation burden

## Abstract

Lung adenocarcinoma (LUAD) is at present the most prevalent subtype of lung cancer worldwide. Non-SMC condensin I complex subunit D2 (NCAPD2) is one of the 3 non-SMC subunits in condensin I. Previous studies have confirmed that NCAPD2 plays a critical role in chromosome cohesion and segregation. NCAPD2 may be involved in tumorigenesis and progression by participating in abnormal cell cycle division, but the prognostic value of NCAPD2 in LUAD remains unclear. We investigated differences in the expression levels of NCAPD2 and determined their association with clinical features, as well as their diagnostic and prognostic value using the cancer genome atlas database. The function of NCAPD2 was analyzed using gene ontology, Kyoto encyclopedia of genes and genomes, and gene set enrichment analysis. CIBERSORT, single-sample gene set enrichment analysis, and ESTIMATE were used to analyze the immune microenvironment of tumor patients. Tumor mutational burden (TMB) and immune checkpoints were analyzed, while hub genes were identified using weighted gene coexpression network analysis and were used to construct prognostic models. Subsequently, the competing endogenous RNAs network of NCAPD2 in LUAD was explored. Finally, we performed qPCR to verify differences in NCAPD2 expression between the tumor and normal tissues. The expression of NCAPD2 in LUAD was significantly upregulated compared with normal lung tissues. NCAPD2 has been linked to the T stage, N stage, and tumor stage. The elevated expression of NCAPD2 in LUAD can predict a poor prognosis. Functional enrichment analysis indicated that the main function of NCAPD2 was in cell cycle regulation. Moreover, NCAPD2 was also associated with immune cell infiltration and TMB. NCAPD2 is a novel prognostic marker in LUAD and is associated with immune infiltration and TMB.

## 1. Introduction

The leading type of cancer that leads to death worldwide is lung cancer.^[1]^ Lung cancer can be divided into 2 main subtypes: small cell lung cancer and non-small cell lung cancer, which account for 15% and 85% of all cases, respectively.^[2]^ Lung adenocarcinoma (LUAD) is currently the most prevalent type of lung cancer worldwide.^[3]^ At present, the 5-year overall survival (OS) rate of lung cancer is not optimistic and is only about 15%, mainly due to the lack of precision and diversification in diagnosis and treatment methods.^[4]^ Therefore, it is necessary to strengthen the exploration of the molecular mechanism of lung cancer and develop more effective early screening and late treatment methods.

Improvements in molecular pathology testing have led to major advances in the treatment of non-small cell lung cancer, along with significant improvements in overall survival as a result of immunotherapy and targeted therapy.^[5]^ LUAD can be treated using EGFR, ALK, and TKI therapy to achieve long-term survival, but resistance to targeted therapy is the main challenge that results in a poor treatment effect.^[6]^ Therefore, more effective biomarkers of LUAD, as well as new molecular mechanisms and therapeutic targets need to be identified. Condensins is a multi-subunit protein complex that is involved in mitotic chromosome assembly and dissociation, and are classified as condensins I and II, respectively based on their function. Structural maintenance of chromosomes (SMC) proteins and 3 non-SMC subunits constitute the condensed I complex, while the D2 subunit (NCAPD2) is one of the non-SMC subunits.^[7]^ NCAPD2 is involved in chromosome structural changes and segregation during mitosis in eukaryotic cells.^[8]^ Chromosomal abnormalities are often associated with tumorigenesis and immune system abnormalities.^[9]^ Recent studies have confirmed that NCAPD2 is overexpressed in breast cancer and colorectal cancer, and is closely associated with prognosis, as a risk factor.^[10,11]^ Moreover, as a hub gene or candidate gene, NCAPD2 is involved in the invasion process of a variety of cancers and is a potential therapeutic target in hepatocellular carcinoma, gastric cancer, and ovarian cancer.^[12–14]^ However, at present, the clinical application and functional mechanisms of NCAPD2 in LUAD remain unclear.

## 2. Materials and methods

### 2.1. Data acquisition

LUAD mRNA count and fpkm data were obtained from the cancer genome atlas (TCGA) database using UCSC Xena^[15]^ (https://xenabrowser.net/), including 510 tumor tissue and 58 normal tissue samples. Corresponding clinical and survival data were also downloaded. The GSE10072 and GSE50081 datasets were acquired from gene expression omnibus (https://www.ncbi.nlm.nih.gov/geo/) database.^[16]^ The GSE50081 dataset included data on 181 NSCLC cases, from among which we selected LUAD data to be used in the analysis. The gene expression omnibus datasets used the GPL96 and GPL570 platforms. We downloaded microRNA (miRNA) data on LUAD from TCGA. The Fasta format sequences of all mature miRNA sequences were obtained from the miRbase (https://www.mirbase.org/). We downloaded the long non-coding RNA (lncRNA) data of LUAD from the UCSC Xena database and then log2-transformed the data. Simple nucleotide variation data of LUAD included in TCGA database were obtained from Genomic Data Commons (https://portal.gdc.cancer.gov/).

### 2.2. Diagnostic value of NCAPD2 in LUAD

TCGA data were extracted to analyze differences in mRNA expression levels of NCAPD2 between the tumor and normal tissues, and the GSE10072 dataset was applied to verify the differences. The diagnostic value of NCAPD2 in LUAD was evaluated by establishing a receiver operator characteristic (ROC) curve. Immunohistochemical staining of the Human Protein Atlas (HPA) (www.proteinatlas.org) was performed to validate the differences in the protein level expression of NCAPD2.

### 2.3. Survival analysis of NCAPD2

To investigate whether NCAPD2 expression affects prognosis, the R packages, survival and survminer, were used to conduct the survival analysis. The tumor cases in TCGA database were divided into 2 groups based on the NCAPD2 expression level (cutoff value was 50%). The Kaplan–Meier (KM) method was applied to construct the survival curve combined with the overall survival time. The GSE50081 dataset was applied for verification.

### 2.4. Identification of the differential expression genes (DEGs) and function analysis of NCAPD2

To identify differential expression genes (DEGs) associated with NCAPD2 expression, the R package, limma, was used to analyze tumor samples obtained from TCGA. The DEG thresholds were set at the absolute value of log fold change(|logFC|) ≥ 1.0. The R packages, ggpubr and ggthemes, were used to construct a volcano map of the DEGs. To explore the functional mechanism of NCAPD2 in LUAD, gene ontology^[15]^ (GO) functional enrichment, Kyoto encyclopedia of genes and genomes^[17]^ (KEGG), and gene set enrichment analysis^[18]^ (GSEA) were performed on the 1365 DEGs identified using the R packages, clusterProfiler and Enrichment plot. The GO and KEGG analysis results were visualized using a bubble chart, and the GSEA shows the top 10 pathways.

### 2.5. Further analysis of the relationship between NCAPD2 and immune infiltration in LUAD

The composition of the tumor microenvironment (TME) of all LUAD samples were assessed using ESTIMATE.^[19]^ The stromal score, immune score, ESTIMATE score, and tumor purity of all the tumor samples were calculated using the R package, estimate, and the differences between the 2 groups of different expression levels of NCAPD2 were analyzed (cutoff value was 50%). CIBERSORT is a deconvolution algorithm that was applied to calculate the proportion of 22 immune cells in all tumor samples. The single-sample gene set enrichment analysis (ssGSEA) quantified the infiltration degree of the 28 subtypes of immune cells in the tumor tissues using the R package, GSVA.

### 2.6. Differences in tumor mutation burden (TMB) and immune checkpoints between the different NCAPD2 expression groups

Since there was a lack of mutational information in some of the LUAD samples, we collected simple nucleotide variation data from 498 patients. The R package, maftools, was used to plot oncoplots for the different NCAPD2 expression groups (cutoff value was 50%). Subsequently, the TMB and immune checkpoint expression of the LUAD samples were compared between the 2 groups.

### 2.7. Identification of hub genes using WGCNA

We performed weighted gene coexpression network analysis (WGCNA)^[20]^ using the R package, WGCNA, to construct a weighted co-expression network and screen modules associated with clinical phenotypes. The 1365 DEGs were divided into different modules by WGCNA, and the correlation between the modules and clinical phenotypes was demonstrated using a heatmap. The modules most associated with the clinical phenotype were identified as key modules. Then, 19 genes were identified from among the key modules as hub genes based on gene significance (GS) and module membership (MM).

### 2.8. Protein-protein interaction (PPI) network and survival analysis of the hub genes

The interactions between the hub genes that encoded proteins were analyzed using the STRING database^[21]^ (https://string-db.org/). The R package, survival, was used to conduct the COX regression analysis of the hub genes, and were visualized using the R package, forestplot. The survival analysis and drawing of the survival curve of the hub genes were performed, and the same were analyzed and visualized using the R packages, survival and survminer, for comparisons between high expression and low expression (cutoff value was 50%).

### 2.9. Construction of a prognostic model

The hub genes were used to perform the LASSO analysis using the R package, glmnet, and the genes most closely associated with prognosis were identified. Based on the expression and regression coefficient of the related genes, the risk score was calculated using the formula. The tumor samples were classified into either the high-risk and low-risk groups, based on the median risk score, and subsequent analyses were performed. The time-dependent ROC curve was drawn based on the risk score.

### 2.10. Exploration of the competing endogenous RNAs (ceRNAs) network of NCAPD2

The competing endogenous RNA (ceRNA) network creates a link between mRNAs and non-coding RNAs to regulate the progression of cancers.^[22]^ To explore the ceRNA network of NCAPD2, we used specific databases for analysis. We identified miRNAs associated with NCAPD2 from the ENCORI database (https://starbase.sysu.edu.cn/), in which we selected the “miRNA-mRNA” option and did not modify any option after entering the same. The correlation between the identified miRNAs and NCAPD2 were analyzed. Based on ceRNA theory, the expression of the predicted miRNAs is inversely correlated with NCAPD2 expression. Therefore, we set the condition as Spearman coefficient r < -.3 and *P* < .05. miRNAs that met the above criteria were analyzed to identify differences, and the miRNAs with a significant difference were analyzed for survival. The miRNAs with differential expression between the tumor and normal tissues and that contributed to the prognosis were considered as core miRNAs of NCAPD2. In the ENCORI Database, we selected the “miRNA-lncRNA” option and selected the input as core miRNAs. The “target” parameter was changed to all, and other conditions remain as default. Based on ceRNA theory, the expression of the predicted lncRNAs was inversely correlated with the expression of the core miRNAs. Therefore, we set the condition as a Spearman coefficient of r < -.3 and *P* value of < .05. The lncRNAs with differential expression between tumor and normal tissues and those that contributed to the prognosis were considered as core lncRNAs. In the survival analysis, the optimal cutoff value was used to divide the tumor samples into 2 groups, high expression and low expression.

### 2.11. Collection and expression validation of cancer and adjacent tissues obtained from LUAD patients

After informed consent was obtained from the patients and approval was obtained from the Ethics Committee of the First Affiliated Hospital of Guangxi Medical University, we collected tumor and lung tissues from 15 LUAD patients (see Figure S1, Supplemental Digital Content, http://links.lww.com/MD/I337, in which approval of the ethical review provided by the agency is presented). Total RNA obtained from these tissues was extracted and reversed transcribed into complementary DNA. qPCR was used to quantitatively analyze NCAPD2 expression using Fast Start Universal SYBR Green Master (Roche, Germany) system. The forward and reverse primer sequences of the NCAPD2 were as follows: NCAPD2-F, 5′-ATGGCTTTGACTGGGAAGAAGAG-3′; NCAPD2-R, 5′-GGCGGTTCTTCTGGTGATTAATG-3′.

### 2.12. Statistical analysis

The statistical analysis was performed using R software, version 4.2.0 (https://cran.r-project.org/). Two-group differential expression analyses were performed using a t-test. The log-rank test was used to perform the KM survival analysis, while COX analysis was used to determine the independence factor of NCAPD2. LASSO analysis was used to construct the prognostic models. The Spearman method was used to determine the correlation analysis. A *P* value of < .05 was used as the significant difference threshold (*P* > .05, ns; *P* < .05, *; *P* < .01, **; *P* < .001, ***).

## 3. Results

### 3.1. Diagnostic value of NCAPD2 in LUAD

**Figure 1.**
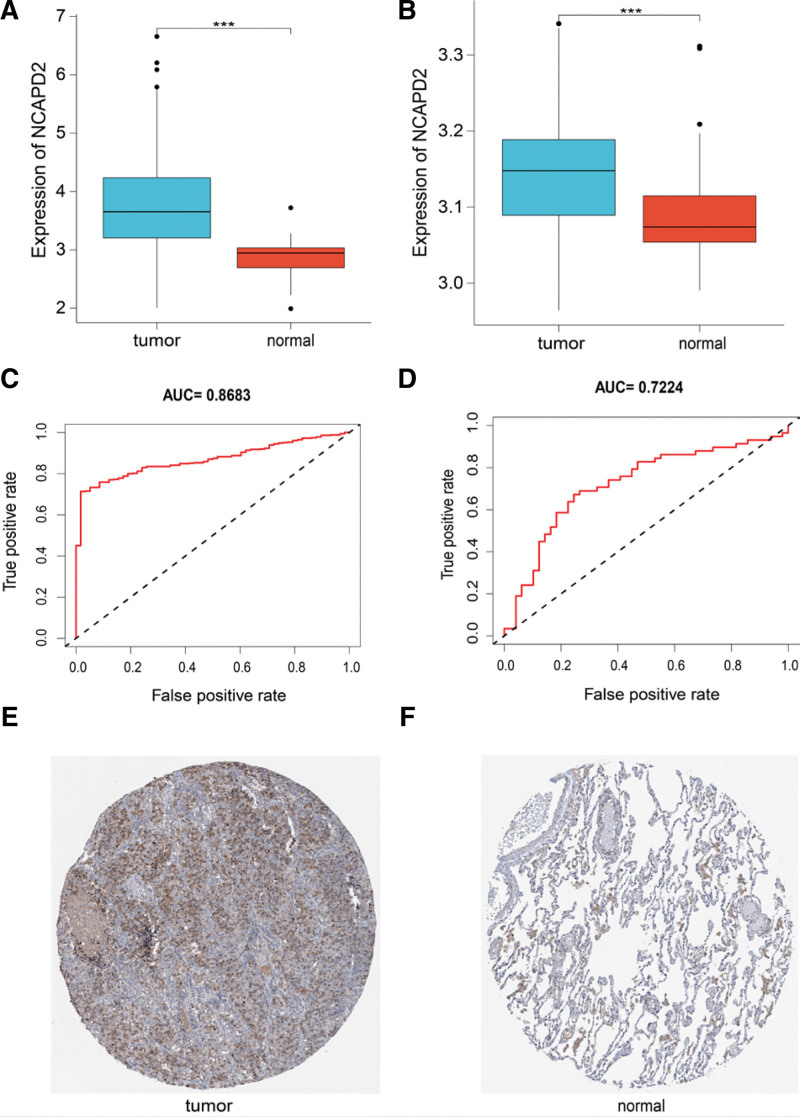
Diagnostic value of NCAPD2 in LUAD. (A) Differential NCAPD2 expression in the tumor and normal groups of TCGA dataset. (B) Differential NCAPD2 expression in the tumor and normal groups of the GSE10072 dataset. (C) Diagnostic ROC curve of NCAPD2 in TCGA dataset. (D) Diagnostic ROC curve of NCAPD2 in the GSE10072 dataset (true positive rate = sensitivity, false positive rate = (1 – specificity)). (E and F) Immunohistochemistry images of NCAPD2 in LUAD obtained from the HPA database. LUAD = lung adenocarcinoma, NCAPD2 = non-SMC condensin I complex subunit D2, ROC = receiver operator characteristic, TCGA = the cancer genome atlas.

Analysis of TCGA dataset showed that NCAPD2 expression was higher in LUAD than in normal lung tissues (Fig. 1A). Analysis of the validation dataset (GSE10072) showed that NCAPD2 was overexpressed in LUAD (Fig. 1B). We used ROC curve analysis to distinguish LUAD from normal lung tissue, and found that the area under the ROC curve from TCGA dataset (Fig. 1C) and GSE10072 dataset (Fig. 1D) were.8683 and.7224, respectively, suggesting that NCAPD2 is a potential diagnostic biomarker. Results of immunohistochemistry assay demonstrated that NCAPD2 was upregulated in LUAD tissues, compared with normal lung tissues (Fig. 1E, F).

### 3.2. Survival and clinical value of NCAPD2

**Figure 2.**
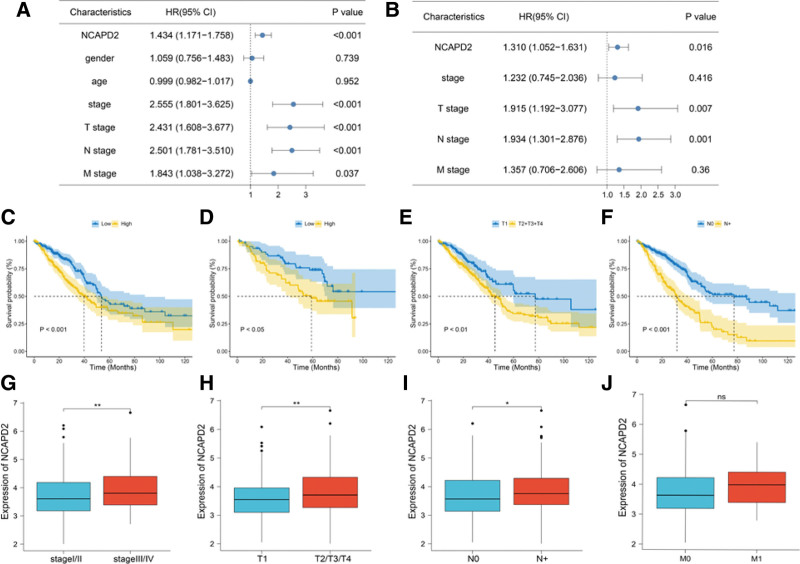
The expression of NCAPD2 in LUAD is valuable for survival and is associated with a variety of clinical characteristics. (A) Univariate COX regression analysis. (B) Multivariate COX regression analysis. (C) KM survival analysis between the 2 groups with different NCAPD2 expression levels in TCGA dataset. (D) KM plot of NCAPD2 in the GSE50081 dataset. (E) KM survival analysis of clinical T stage. (F) KM survival analysis of clinical N stage. (G) Comparison of NCAPD2 expression in LUAD between the different pathological stages. (H) NCAPD2 expression was associated with clinical T stage in LUAD. (I) NCAPD2 expression was associated with clinical N stage in LUAD. N+: N1, N2, N3 stage. (J) Comparison of NCAPD2 expression at different clinical M stages. HR = hazard ratio, KM = Kaplan–Meier, LUAD = lung adenocarcinoma, NCAPD2 = non-SMC condensin I complex subunit D2, TCGA = the cancer genome atlas.

The COX univariate analysis (Fig. 2A) and multivariate analysis (Fig. 2B) indicated that NCAPD2 could be used as an independent prognostic factor to predict poor prognosis. KM survival analysis results of TCGA (Fig. 2C) and GSE50081 (Fig. 2D) datasets indicated that high NCAPD2 expression can predict a poor prognosis of LUAD. Tumor cases with high clinical T stage (Fig. 2E) or N stage (Fig. 2F) showed a poor prognosis. NCAPD2 expression was upregulated in patients with an advanced pathological stage (Fig. 2G). NCAPD2 expression tended to be higher in groups with a high clinical T stage in LUAD (Fig. 2H). NCAPD2 was overexpressed in LUAD patients with lymph node metastasis (N+) (Fig. 2I). NCAPD2 expression showed no significant difference in M stage patients (Fig. 2J).

### 3.3. Functional enrichment analysis of NCAPD2

**Figure 3.**
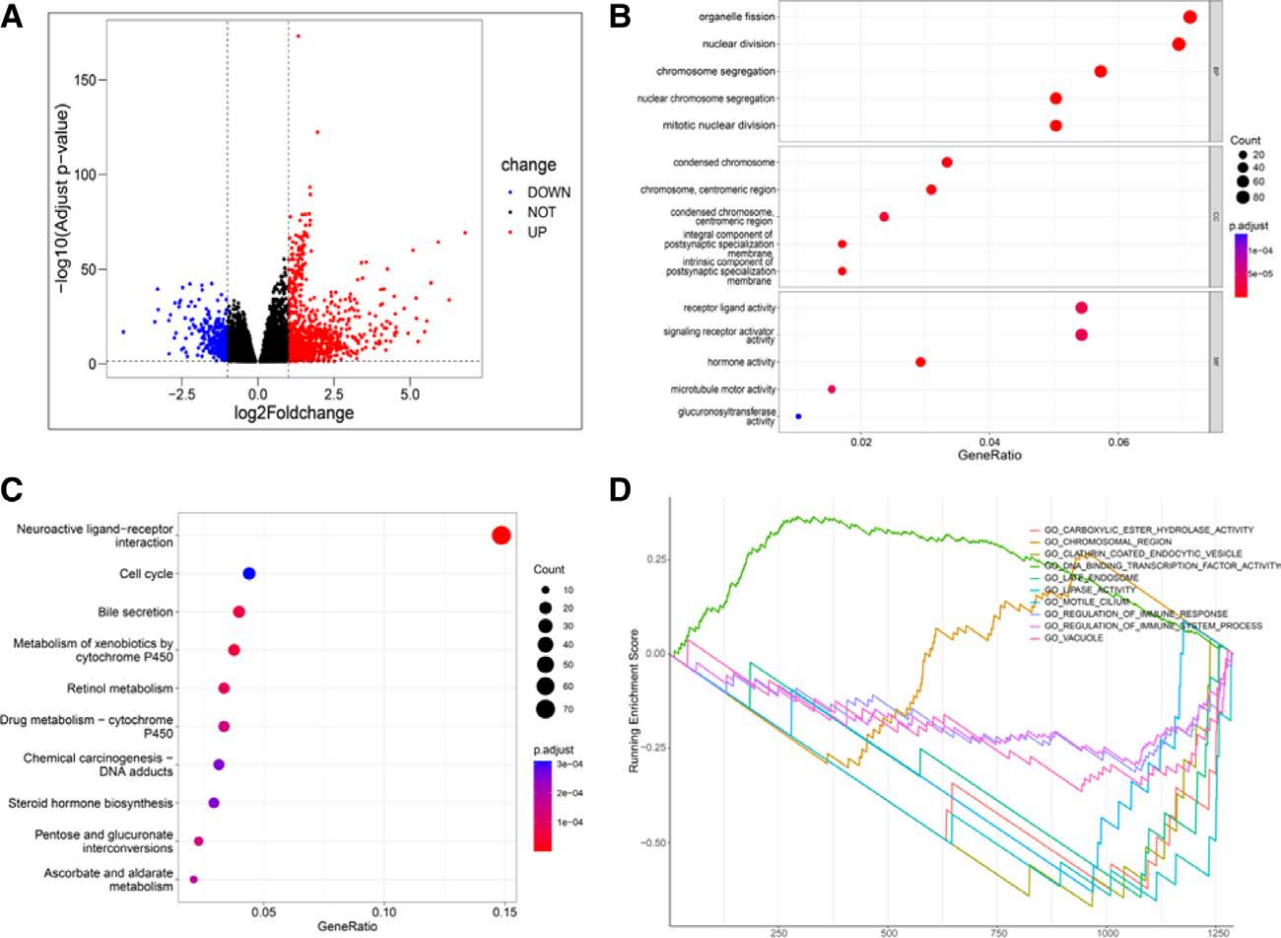
Potential mechanisms of NCAPD2 in LUAD. (A) A volcano map showing the significant DEGs identified through the analysis of differences in NCAPD2 expression in the LUAD cohort. (B) The results of the GO analysis with significant DEGs are shown in the dot plot. (C) The results of the KEGG analysis of significant DEGs are shown in the dot plot. In the dot plot, the size of the dot shows the degree of gene enrichment, and the color of the dot indicates significance. (D) Presentation of the results of the GSEA enrichment analysis of the DEGs. DEG = differential expression genes, GO = gene ontology, GSEA = gene set enrichment analysis, KEGG = Kyoto encyclopedia of genes and genomes, LUAD = lung adenocarcinoma, NCAPD2 = non-SMC condensin I complex subunit D2.

A total of 1365 DEGs were obtained by analyzing the mRNA expression profiles of the 2 groups with different NCAPD2 expression in the LUAD cohort (Fig. 3A) and were subjected to functional enrichment analysis. All biological processes (BP) in the GO analysis were associated with the cell cycle, including “organelle fission,” “nuclear division,” “chromosome segregation,” “nuclear chromosome segregation,” and “mitotic nuclear division” (Fig. 3B). In the KEGG enrichment analysis, the DEGs were enriched in these terms: “Neuroactive ligand − receptor interaction,” “Cell cycle,” “Bile secretion,” “Metabolism of xenobiotics by cytochrome P450,” “Retinol metabolism,” “Drug metabolism − cytochrome P450,” “Chemical carcinogenesis − DNA adducts,” “Steroid hormone biosynthesis,” “Pentose and glucuronate interconversions,” and “Ascorbate and aldarate metabolism,” which are mainly associated with metabolism, cell cycle, and carcinogenesis (Fig. 3C). In the GSEA enrichment analysis (Fig. 3D), the DEGs were found to be enriched in these terms: “GO_CARBOXYLIC_ESTER_HYDROLASE_ACTIVITY,” “GO_CHROMOSOMAL_REGION,” “GO_CLATHRIN_COATED_ENDOCYTIC_VESICLE,” “GO_DNA_BINDING_TRANSCRIPTION_FACTOR_ACTIVITY,” “GO_LATE_ENDOSOME,” “GO_LIPASE_ACTIVITY,” “GO_MOTILE_CILIUM,” “GO_REGULATION_OF_IMMUNE_RESPONSE,” “GO_REGULATION_OF_IMMUNE_SYSTEM_PROCESS,” and “GO_VACUOLE.”

### 3.4. Further analysis of the relationship between NCAPD2 and immune infiltration in LUAD

**Figure 4.**
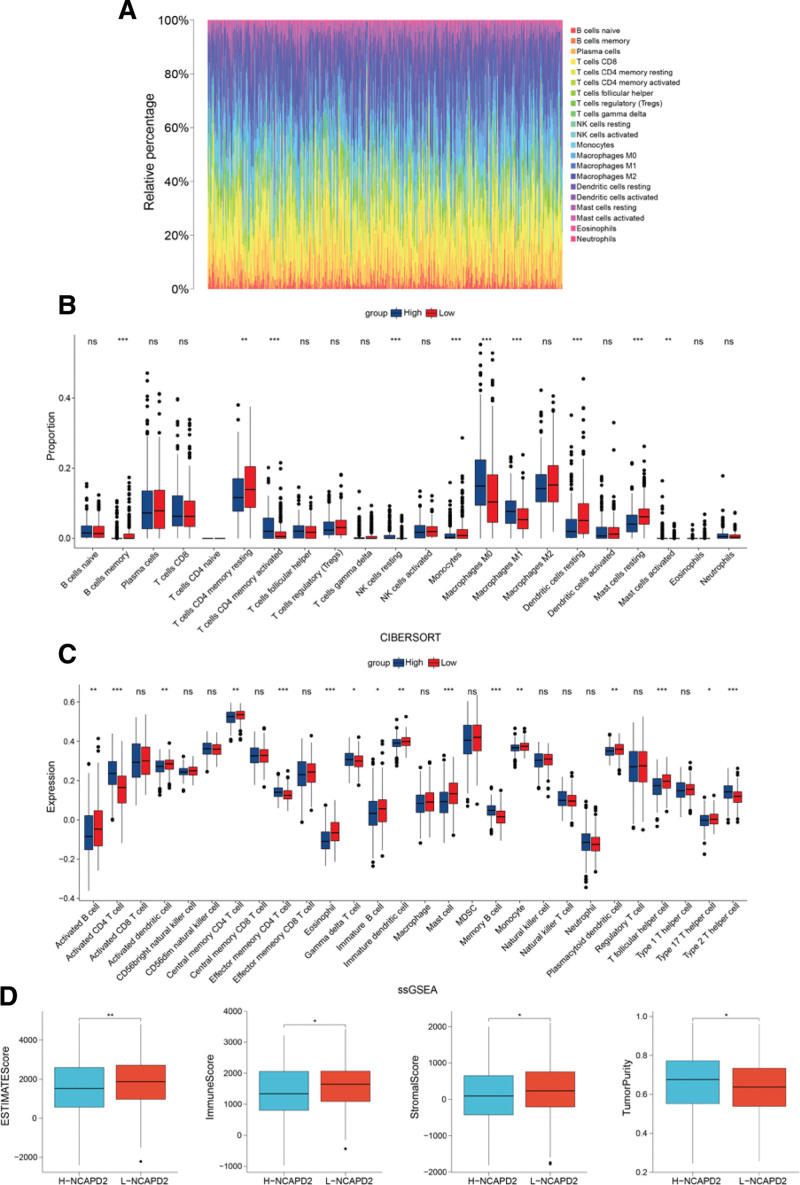
Immune correlation analysis of NCAPD2. (A) Bar plot showing the percentage of the 21 immune infiltrating cells in the LUAD samples. (B) Differences in the proportion of immune cells between the 2 groups with different NCAPD2 expression levels. (C) Comparison of immune cell expression between the 2 groups with different NCAPD2 expression levels. (D) Difference analysis of the ESTIMATE score, immune score, stromal score, and tumor purity between the high and low NCAPD2 expression groups in the LUAD samples. LUAD = lung adenocarcinoma, NCAPD2 = non-SMC condensin I complex subunit D2.

The percentage of immune infiltrating cells in the LUAD patients was calculated and presented using CIBERSORT (Fig. 4A). Furthermore, the CIBERSORT results indicated a positive correlation between NCAPD2 expression and the 5 subtypes of Tumor-infiltrating immune cells (TIICs), including T cells CD4 memory activated, NK cells resting, Macrophages M0, Macrophages M1, and Mast cells activated. NCAPD2 expression showed an inverse correlation with 5 subtypes of TIICs, including B cells memory, T cells CD4 memory resting, Monocytes, Dendritic cells resting, and Mast cells resting (Fig. 4B). The results of the single-sample gene set enrichment analysis ssGSEA showed a positive correlation between NCAPD2 expression and the expression of the 5 subtypes of TIICs, including Activated CD4 T cells, Effector memory CD4 T cells, Gamma delta T cells, Memory B cells, and Type 2 T helper cells. NCAPD2 expression showed an inverse correlation with the expression of eleven subtypes of TIICs, including Activated B cells, Activated dendritic cells, Central memory CD4 T cells, Eosinophils, Immature B cells, Immature dendritic cells, Mast cells, Monocytes, Plasmacytoid dendritic cells, T follicular helper cells, and Type 17 T helper cells (Fig. 4C). The high NCAPD2 expression group had a lower ESTIMATE score, immune score, and Stromal score, but higher tumor purity (Fig. 4D).

### 3.5. Differences in the TMB and immune checkpoints between the different NCAPD2 expression groups

**Figure 5.**
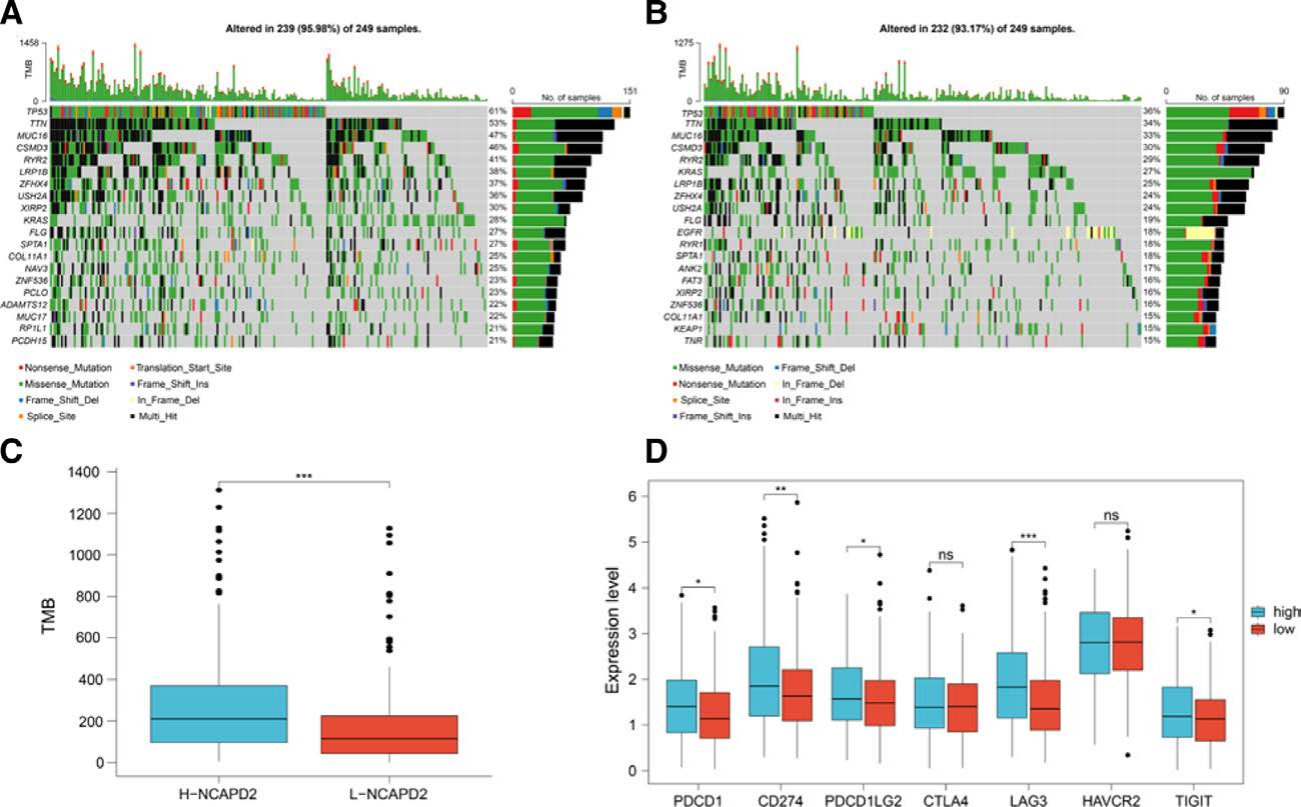
Comparison of gene mutations and exploration of immune checkpoints. (A) Mutation landscape of the high NCAPD2 expression group in LUAD. (B) Mutation landscape of the low NCAPD2 expression group in LUAD. (C) Differential analysis of TMB between the high and low NCAPD2 expression groups in LUAD. (D) Difference analysis of immune checkpoint expression between the high and low NCAPD2 expression groups. LUAD = lung adenocarcinoma, NCAPD2 = non-SMC condensin I complex subunit D2, TMB = tumor mutational burden.

Immune checkpoint expression and gene mutations can affect the efficacy of immunotherapy for tumors. The gene mutation map of the 2 groups with different NCAPD2 expression levels is shown with the top 20 mutated genes indicated in Fig. 5A, B. In LUAD, NCAPD2 high expression group showed a higher TMB than the NCAPD2 low expression group (Fig. 5C). In the analysis of the high and low NCAPD2 expression groups, the expression levels of 5 immune checkpoints, including PDCD1, CD274, PDCD1LG2, LAG3, and TIGIT, were higher in the NCAPD2 high expression group (Fig. 5D).

### 3.6. Identification of hub genes using WGCNA

**Figure 6.**
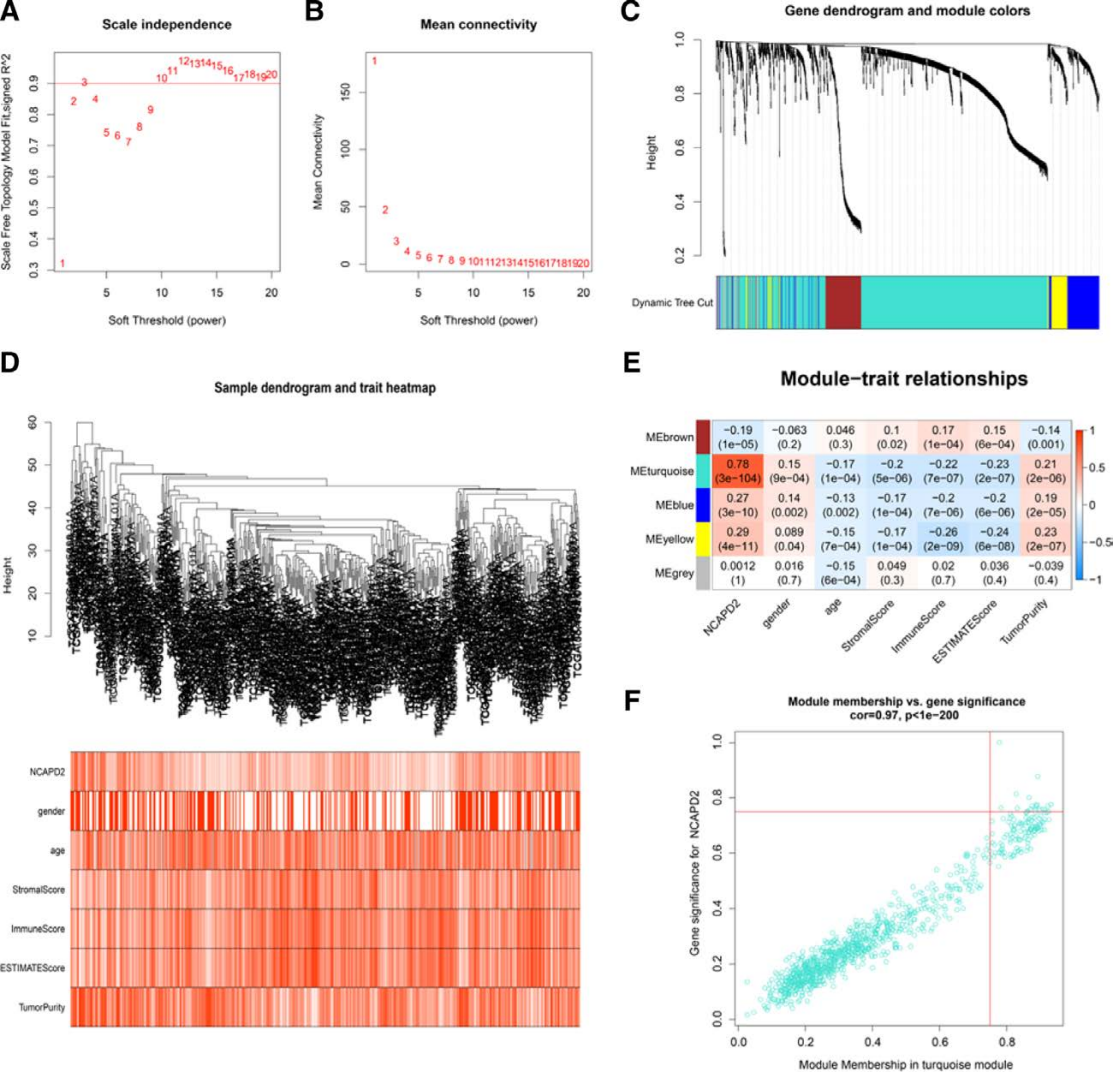
Identification of hub genes in LUAD. (A) the scale-free fitting index was calculated through diverse soft thresholds (power). (B) Based on diverse soft thresholds (power), the mean connectivity was analyzed. (C) Gene dendrogram showing the clustering of the LUAD samples. (D) The composite graph showed the clustering of the LUAD samples and the correlation of clinical parameters. (E) Heatmap between the gene modules and clinical parameters. (F) Scatter plot of the turquoise module eigengenes. LUAD = lung adenocarcinoma.

To identify hub genes, the 1365 DEGs were subjected to WGCNA analysis. After adjusting for a series of parameters, the average link hierarchical clustering method was adopted to cluster the DEGs into 5 modules (Fig. 6A–D). The heatmap indicated that the turquoise module had the maximal Pearson’s correlation coefficient with NCAPD2 expression, and this module contained 947 genes (Fig. 6E). The 19 genes in the turquoise module were identified as hub genes as module membership and gene significance were both > 0.75 (Fig. 6F).

### 3.7. PPI network and survival analysis of the hub genes

**Figure 7.**
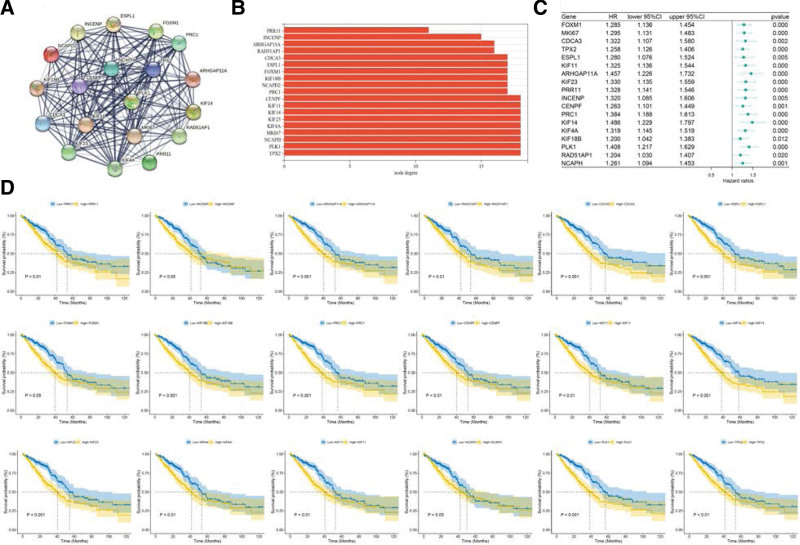
The interaction network of the hub genes and further studies. (A) Construction of the PPI network of the hub genes. (B) The bar chart shows the node degrees of the PPI network. (C) The forest plot of the COX analysis of hub gene expression. (D) Kaplan–Meier survival analysis of the hub genes. PPI = protein–protein interaction.

The PPI network of the hub genes was constructed using the STRING database, and the node degrees were between 11 and 18 (Figs. 7A, B). The COX analysis indicated that all hub genes were risk factors (HR > 1) (Fig. 7C) that contributed to a poor prognosis, according to the KM survival analysis (Fig. 7D). One of the hub genes, COX analysis, and survival analysis of NCAPD2 are presented in Figure 2.

### 3.8. Construction of prognostic model

**Figure 8.**
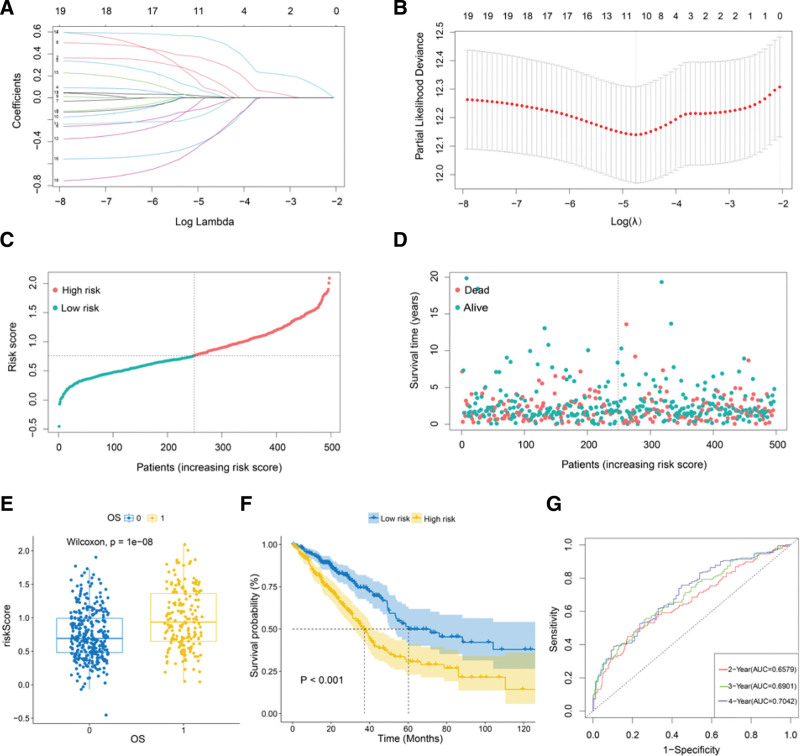
Construction of the prognostic model. (A-B) LASSO analysis of the hub genes. (C) Scatterplot of the risk scores ranked from lowest to highest. (D) Scatterplot of the samples based on survival time, survival status, and risk scores. (E) Difference analysis of the risk scores among samples with different survival statuses. 0: alive, 1: dead. (F) KM survival analysis between the high and low risk groups. (G) ROC curves for 2, 3, and 4 years in the prognostic model.

Complete information was available on a total of 497 LUAD samples and were analyzed. The LASSO analysis was performed using the expression matrix of the 19 hub genes identified in the LUAD patients, and the 10 most relevant genes were identified based on the optimum λ value (Fig. 8A, B). The risk score was transformed using the expression and coefficients of the 10 genes: risk score = (.176931458851421 * FOXM1) + (.0491298316698072 * TPX2) + (-.0820432513523308 * ESPL1) + (.342132744093238 * ARHGAP11A) + (-.132199951912977 * INCENP) + (.0130539838947839 * PRC1) + (.0804159890158544 * KIF14) + (-.373151954809323 * KIF18B) + (.536526888492945 * PLK1) + (-.377874611444398 * RAD51AP1). The tumor patients were labeled as high or low risk based on a median risk score and assigned to 2 different groups (Fig. 8C). The survival status of each patient (on the left side of the dotted line: low-risk population; on the right side of the dotted line: high-risk population) (Fig. 8D). Then, we compared risk scores of the risk model, and the results showed that the risk scores of the death group were significantly higher (Fig. 8E), and that the prognosis of the high-risk group was poor (Fig. 8F). The area under ROC curves of this model at 2, 3 and 4 years were 0.6579, 0.6901, and 0.7042 (Fig. 8G).

### 3.9. Exploration of the ceRNA network

**Figure 9.**
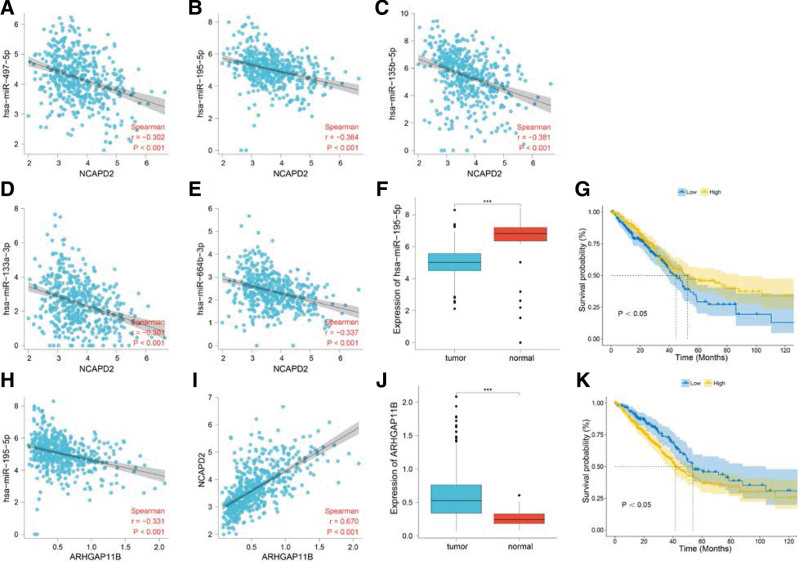
Construction of the ceRNA network for NCAPD2. (A-E) Correlation analysis between the 5 predicted miRNAs and NCAPD2. (F) Difference analysis of hsa-miR-195-5p expression in the LUAD and lung tissues. (G) Survival analysis of hsa-miR-195-5p in the LUAD samples. (H) Correlation analysis between hsa-miR-195-5p and the lncRNA predicted by hsa-miR-195-5p. (I) Correlation analysis between NCAPD2 and the lncRNA predicted by hsa-miR-195-5p. (J) Differential analysis of ARHGAP11B expression in LUAD and lung tissues. (K) Survival analysis of ARHGAP11B in the LUAD samples. ceRNA = competing endogenous RNA, LUAD = lung adenocarcinoma, NCAPD2 = non-SMC condensin I complex subunit D2.

The 5 predicted miRNAs showed an inverse correlation with NCAPD2 expression (Fig. 9A–E). Among the 5 predicted miRNAs, hsa-miR-195-5p was overexpressed in lung tissues (Fig. 9F), while its overexpression in tumor samples predicted a good prognosis (Fig. 9G). Therefore, hsa-miR-195-5p was selected from among the 5 miRNAs as the core miRNA. ARHGAP11B was the lncRNA predicted by hsa-miR-195-5p, and showed a negative correlation (Fig. 9H). The expression of ARHGAP11B and NCAPD2 were positively correlated (Fig. 9I). ARHGAP11B expression was higher in tumor tissues (Fig. 9J), and its overexpression in the tumor samples predicted a poor prognosis (Fig. 9K). Therefore, ARHGAP11B was identified as the core lncRNA.

### 3.10. Validation of NCAPD2 expression in LUAD and adjacent lung tissues

**Figure 10.**
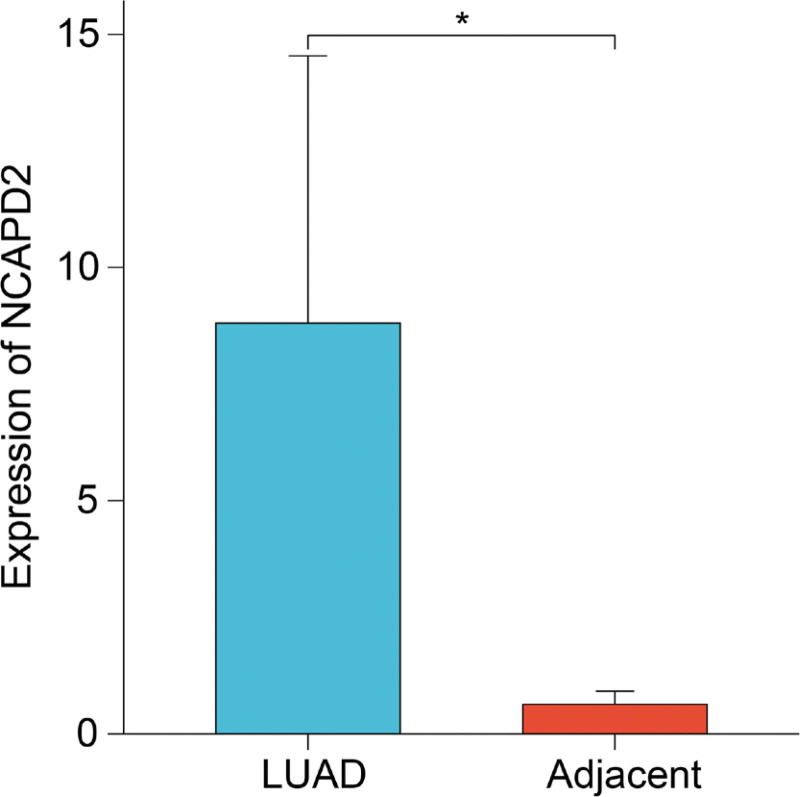
Validation of NCAPD2 expression using RT-qPCR in fresh tumor and lung tissues obtained from LUAD patients. LUAD = lung adenocarcinoma, NCAPD2 = non-SMC condensin I complex subunit D2.

We determined NCAPD2 expression levels in fresh tumors and adjacent tissues of 15 LUAD patients using RT-qPCR to confirm the overexpression of NCAPD2 in LUAD tissues (Fig. 10).

## 4. Discussion and Conclusions

Conclusively, we found that NCAPD2 was overexpressed in LUAD samples, and that NCAPD2 expression could be used to distinguish LUAD from normal lung tissue. The GEO and HPA databases were used to confirm the difference in NCAPD2 expression between tumor and normal tissues. The analysis of results obtained from the above 3 databases proved that NCAPD2 may be a potential diagnostic marker for LUAD.

KM survival analysis showed that NCAPD2 overexpression predicted a poor prognosis of LUAD patients. COX multivariate regression analysis indicated that NCAPD2 may be an independent prognostic factor for LUAD. In addition, NCAPD2 expression was closely associated with T, N, and pathological stage. The overexpression of NCAPD2 in the LUAD samples suggested tumor progression and invasion, which further indicated that NCAPD2 expression was inversely correlated with prognosis.

We investigated the molecular function of NCAPD2 in LUAD through GO, KEGG, and GSEA analyses. It has been documented that the occurrence and progression of tumors are associated with an abnormal cell cycle.^[23]^ The GO and KEGG analyses suggested that NCAPD2 expression was associated with the cell cycle, which may suggest that NCAPD2 promotes tumor progression in LUAD by mediating the cell cycle. In the KEGG enrichment analysis, NCAPD2 was found to be associated with P450 and Chemical carcinogenesis, while P450 contributes to the activation of carcinogens and carcinogenesis.^[24]^ The GSEA enrichment analysis suggested that NCAPD2 was associated with immune regulation. The results of the above mentioned functional analysis suggested that NCAPD2 exerts an indispensable role for the occurrence and development of LUAD.

The TME is associated with tumor occurrence and progression. Based on the previous functional analysis, we further analyzed interactions between NCAPD2 and tumor immune infiltration in LUAD. Approximately 40% of B cells in adults are memory B cells, and the production of memory B cells is one of the key features of adaptive immunity.^[25]^ One study showed that B cells, a type of antigen-presenting cell, can induce anti-tumor immunity and produce antibodies.^[26]^ CD4 T cells are closely associated with the antitumor response, which can exert indirect antitumor effects by enhancing the antitumor activity of other antitumor effector cells and direct antitumor effects by producing effector cytokines, such as tumor necrosis factor-α (TNFα) and interferon-γ (IFNγ).^[27]^ Monocytes play various roles during each stage of cancer, including antitumor and tumor-promoting effects.^[28]^ Dendritic cells (DC) can infiltrate tumors and have antigen presentation functions, and participate in anti-tumor T-cell immunity.^[29]^ Mast cells have anti-tumor or tumor-promoting effects, which mainly depend on the environment, and play an anti-tumor role in lung cancer.^[30]^ T follicular helper cells increase the antitumor effect by promoting cytokine production and cytotoxic function in exhausted T cells and exert antitumor immunity in a CD8(+) -dependent manner.^[31]^ Th17 cells are indirectly involved in antitumor effects by promoting T cell recruitment to tumors and CD8 + T cell priming.^[32]^ In this study, CIBERSORT and ssGSEA analyses showed that NCAPD2 overexpression in LUAD cases was associated with low infiltration levels of the immune cells mentioned above. In the ESTIMATE analysis, LUAD samples with high NCAPD2 expression showed higher tumor purity and a lower infiltration level of immune cells and stromal cells, compared with those with low NCAPD2 expression. Based on the above study on the immune environment of the LUAD samples, the high NCAPD2 expression group had lower immune cell infiltration and poorer immune ability.

The use of immune checkpoint inhibitors (ICIs) has become the main method of immunotherapy for cancer, and can improve the survival rate of lung cancer patients and provides an effective method of treatment for advanced lung cancer.^[33]^ In the gene mutation analysis, LUAD patients with high NCAPD2 expression had a higher TMB than LUAD patients with low NCAPD2 expression. TMB may modulate the response of cancer patients to ICIs by affecting the production of immunogenic peptides, and a significant association between high TMB and ICI treatment has been found in a variety of tumors.^[34]^ In the LUAD samples, 5 immune checkpoints, including PDCD1, CD274, PDCD1LG2, LAG3, and TIGIT, showed high expression in the high NCAPD2 expression group. The combination of PDCD1 (PD-1) and CD274 (PD-L1) may lead to the destruction of the immune environment, resulting in a reduction of T cell excitability and even T cell depletion, as well as a reduction of TNF, IFN-γ, and other cytokines, so as to achieve tumor immune escape.^[35]^ PDCD1LG2 (PD-L2) is the second ligand of PD-1, and binding to PD-1 can also inhibit T cell activation and cause tumor cells to evade immune response.^[36]^ LAG3 can reduce the proliferation of T cells and the secretion of certain cytokines, which leads to the depletion of CD8 + T cells and anti-tumor immune failure.^[37]^ TIGIT can impair the body’s anti-tumor immune response by causing the dysfunction of T cells and natural killer cells.^[38]^ Compared with monotherapy, anti-PD-1/anti-LAG3 or anti-PD-1/anti-TIGIT combined immunotherapy may exert a better inhibition effect of tumor growth.^[38,39]^ Therefore, the LUAD group with high NCAPD2 expression may be more suitable for immunotherapy to reestablish immune function and benefit from immunotherapy.

The 19 hub genes identified using WGCNA are PRR11, INCENP, ARHGAP11A, RAD51AP1, CDCA3, ESPL1, FOXM1, KIF18B, NCAPD2, PRC1, CENPF, KIF11, KIF14, KIF23, KIF4A, MKI67, NCAPH, PLK1, and TPX2. The associated PPI network was constructed, and the results indicated that the 19 hub genes closely interacted with each other. The COX regression analysis and survival analysis showed that 19 hub genes were factors that indicated a poor prognosis and promoted tumor progression in LUAD. Based on previous studies, in addition to NCAPD2, 18 hub genes have also been reported to be overexpressed in lung cancer and contribute to a poor prognosis.^[40–58]^ Subsequently, we constructed a prognostic model, which exerted a good predictive effect. The above studies further demonstrated that hub genes, including NCAPD2, jointly promote the occurrence and progression of LUAD.

In the ceRNA network, miRNAs can inhibit the production of proteins during the translation process by binding to mRNAs, and lncRNAs acts as a sponge for miRNAs to reduce the inhibitory effect of miRNAs on mRNAs, so as to regulate the progression of tumors.^[59,60]^ The upstream miRNAs of NCAPD2 were predicted, and 5 miRNAs were obtained. hsa-miR-195-5p was identified as the core miRNA of NCAPD2 in LUAD due to its statistical significance in both the differential analysis and survival analysis. Relevant studies have reported that hsa-miR-195-5p can be used as a diagnostic marker for lung cancer.^[61]^ hsa-miR-195 can inhibit lung cancer and contributes to a favorable prognosis.^[62]^ The upstream lncRNA predicted by hsa-miR-195-5p was ARHGAP11B, which was identified as the core lncRNA.

In conclusion, NCAPD2 may be an emerging marker for LUAD that can be used for diagnosis and to predict prognosis and is associated with clinical stage. We found that NCAPD2 may promote the progression of LUAD by affecting the cell cycle and immune-related pathways. Although we conducted a comprehensive analysis of NCAPD2 and cross-validation of multiple datasets, cellular experiments were not conducted to verify the results of this study. Therefore, the therapeutic value and function of NCAPD2 in LUAD should be further investigated.

## Acknowledgments

We would like to thank public databases, such as TCGA and GEO for their contribution to the progression of human medicine.

## Author contributions

**Conceptualization:** Zihao Li, Mingwu Chen.

**Data curation:** Yuxuan Zheng, Zuotao Wu.

**Formal analysis:** Zihao Li, Ting Zhuo.

**Investigation:** Zuotao Wu, Yongjie Zhu.

**Methodology:** Zihao Li, Yongyong Wang, Mingwu Chen.

**Project administration:** Mingwu Chen.

**Software:** Yuxuan Zheng.

**Writing – original draft:** Zihao Li, Mingwu Chen.

**Writing – review & editing:** Zihao Li, Lei Dai, Mingwu Chen.

## Supplementary Material
