# Supplementary material for: NCAPD2 is a novel marker for the poor prognosis of lung adenocarcinoma and is associated with immune infiltration and tumor mutational burden
Source: Medicine (Baltimore). 2023 Jan 20;102(3):e32686. doi: 10.1097/MD.0000000000032686 (PMC9857258; doi:10.1097/MD.0000000000032686)
Supplement: Supplementary file 1 [file medi-102-e32686-s001.pdf]

广西医科大学第一附属医院
THE FIRST AFFILIATED HOSPITAL OF
GUANGXI MEDICAL UNIVERSITY

FIRST AFFILIATED HOSPITAL of GUANGXI MEDICAL UNIVERSITY
ETHICAL REVIEW COMMITTEE
Approval Notice

Approval Number: 2022-E376-01
Title: Comprehensive analysis of clinical application and bioinformatics mechanism of NCAPD2 in lung adenocarcinoma
Research Contents: Lung adenocarcinoma (LUAD) is currently the most prevalent subtype of lung cancer worldwide. Non-SMC condensin I complex subunit D2 (NCAPD2) is one of the three non-SMC subunits in condensin I. According to previous studies, it has been confirmed that NCAPD2 plays a critical role in chromosome cohesion and segregation. NCAPD2 may be involved in tumorigenesis and progression by participating in abnormal cell cycle division, but the prognostic value of NCAPD2 in LUAD remains unclear. By accessing the Cancer Genome Atlas (TCGA) database, We aimed to explore NCAPD2 and its relationship with LUAD. We investigated differences in NCAPD2 expression levels using public databases and assessed their association with clinical features, diagnostic, and prognostic value. The function of NCAPD2 was analyzed by Gene Ontology (GO) function enrichment, Kyoto Encyclopedia of Genes and Genomes (KEGG) and Gene Set Enrichment Analysis (GSEA). CIBERSORT, Single-sample Gene Set Enrichment Analysis (ssGESA), and ESTIMATE were used to analyze the immune microenvironment of tumor cases, and immunotherapy was explored using tumor mutational burden (TMB) and immune checkpoints. hub genes were identified using weighted gene coexpression network analysis (WGCNA) and used to construct Protein-Protein Interaction (PPI) networks and prognostic models. Finally, competing endogenous RNAs (ceRNAs) network of NCAPD2 in LUAD was explored. We tested NCAPD2 expression levels in fresh tumors and adjacent tissues from 15 LUAD patients by RT-qPCR,and to verify the difference of NCAPD2 expression between tumors and normal lung tissues
Applicant: Mingwu Chen
Application Department: Department of Cardio-Thoracic Surgery
Date of Application: November 02, 2022
Date of Approval: November 03, 2022
Conclusion: This paper fully considered and protected the rights and interests of the study objects. It meets the criteria of Ethical Review Committee. The Medical Ethics Committee of First Affiliated Hospital of Guangxi Medical University has approved the protocol.

Signature: ___ [illegible]

(Vice) Director of Ethical Review Committee
First Affiliated Hospital of Guangxi Medical University

Date: November 03, 2022
